# Convergent and divergent genes expression profiles associated with brain-wide functional connectome dysfunction in deficit and non-deficit schizophrenia

**DOI:** 10.1038/s41398-024-02827-w

**Published:** 2024-02-27

**Authors:** Chao Zhou, Xiaowei Tang, Miao Yu, Hongying Zhang, Xiaobin Zhang, Ju Gao, Xiangrong Zhang, Jiu Chen

**Affiliations:** 1 Department of Geriatric Psychiatry, Affiliated Nanjing Brain Hospital, Nanjing Medical University, Nanjing, Jiangsu China; 2 Department of Psychiatry, Affiliated WuTaiShan Hospital of Medical College of Yangzhou University, Yangzhou, Jiangsu China; 3 Department of Neurology, Affiliated Nanjing Brain Hospital, Nanjing Medical University, Nanjing, Jiangsu China; 4 Department of Radiology, Subei People’s Hospital of Jiangsu Province, Yangzhou University, Yangzhou, Jiangsu China; 5 Institute of Mental Health, Suzhou Psychiatric Hospital, The Affiliated Guangji Hospital of Soochow University, Suzhou, Jiangsu China; 6 Department of Radiology, Nanjing Drum Tower Hospital, The Affiliated Hospital of Nanjing University Medical School, Nanjing, China; 7 Institute of Medical Imaging and Artificial Intelligence, Nanjing University, Nanjing, Jiangsu China; 8 Medical Imaging Center, the Affiliated Drum Tower Hospital, Medical School of Nanjing University, Nanjing, Jiangsu China

**Keywords:** Schizophrenia, Clinical genetics

## Abstract

Deficit schizophrenia (DS) is a subtype of schizophrenia characterized by the primary and persistent negative symptoms. Previous studies have identified differences in brain functions between DS and non-deficit schizophrenia (NDS) patients. However, the genetic regulation features underlying these abnormal changes are still unknown. This study aimed to detect the altered patterns of functional connectivity (FC) in DS and NDS and investigate the gene expression profiles underlying these abnormal FC. The study recruited 82 DS patients, 96 NDS patients, and 124 healthy controls (CN). Voxel-based unbiased brain-wide association study was performed to reveal altered patterns of FC in DS and NDS patients. Machine learning techniques were used to access the utility of altered FC for diseases diagnosis. Weighted gene co-expression network analysis (WGCNA) was employed to explore the associations between altered FC and gene expression of 6 donated brains. Enrichment analysis was conducted to identify the genetic profiles, and the spatio-temporal expression patterns of the key genes were further explored. Comparing to CN, 23 and 20 brain regions with altered FC were identified in DS and NDS patients. The altered FC among these regions showed significant correlations with the SDS scores and exhibited high efficiency in disease classification. WGCNA revealed associations between DS/NDS-related gene expression and altered FC. Additionally, 22 overlapped genes, including 12 positive regulation genes and 10 negative regulation genes, were found between NDS and DS. Enrichment analyses demonstrated relationships between identified genes and significant pathways related to cellular response, neuro regulation, receptor binding, and channel activity. Spatial and temporal gene expression profiles of *SCN1B* showed the lowest expression at the initiation of embryonic development, while *DPYSL3* exhibited rapid increased in the fetal. The present study revealed different altered patterns of FC in DS and NDS patients and highlighted the potential value of FC in disease classification. The associations between gene expression and neuroimaging provided insights into specific and common genetic regulation underlying these brain functional changes in DS and NDS, suggesting a potential genetic-imaging pathogenesis of schizophrenia.

## Introduction

Schizophrenia (SCZ) is a severe mental disorder that affects ~1% of the global population [1]. Among these individuals, about one-third experience poor social functioning due to severe negative symptoms [2, 3]. Research on negative symptoms aims to improve the long-term prognosis of individuals with schizophrenia. However, the severity and clinical stability of negative symptoms vary at different stages of the disorder, making it challenging to obtain consistent research results [4]. To address this issue, Carpenter et al. [5] and Kirkpatrick et al. [6] proposed the concept of deficit schizophrenia (DS) based on the source and duration of negative symptoms, in an effort to enhance the homogeneity of negative symptoms.

DS is characterized by the primary and persistent negative symptoms, and is regarded as a clinically homogeneous subgroup of SCZ [5, 7, 8]. Previous studies have shown that patients with DS differ from those with non-deficit schizophrenia (NDS) in various clinical aspects, such as risk factors, premorbid functioning, disease course, neurobiological correlates, and treatment response [9–13]. These studies suggest that DS may represent a distinct disease entity within SCZ compared to NDS [7, 8]. However, underlying mechanism behind the differential manifestations between DS and NDS is still unclear.

Research on neuroimaging alterations may provide valuable insights into the pathogenesis and diagnostic classification of DS. Functional magnetic resonance imaging (fMRI) techniques, such as Blood Oxygenation Level Dependent (BOLD) fMRI, have allowed researchers to gain a deeper understanding of neural activity and intrinsic neural function networks. Numerous neuroimaging studies have identified brain function alterations associated with cognitive impairments, symptom severity, and treatment effects in patients with SCZ [14–18]. Similarly, studies on DS have reported distinct abnormal functional changes compared to NDS [19], particularly in brain networks such as the default mode network [20], salience network [21], and nucleus accumbens network [22]. Many of these studies have also demonstrated specific associations between alterations in functional connectivity (FC) and negative symptoms and cognitive impairments in DS. These findings suggest that altered brain function plays a crucial role in the pathogenesis of DS.

As is well-known, SCZ has a heritability ranging from 60 to 80%, largely attributable to common risk alleles [23]. Many studies investigating the genetics of SCZ have been published, revealing the potential genetic pathways underlying the disorder [24, 25]. Previously, we have demonstrated the epigenetic features and gene expression of *MMP9* [26] and *CXCL1* [27], and further explored their associations with clinical features in DS and NDS. Given the impact of genetic inheritance on the functional and structural phenotypes of the brain, researchers have shifted focus to genetic-imaging studies of SCZ. Several studies have reported reliable relationships between genetic profiles and neuroimaging measurements in SCZ. For example, studies on structural imaging have reported potential associations between changes in gray matter volume (GMV) and expression of SCZ-related risk genes [28, 29]. Luo et al. [30] explored the SCZ-related genetic-brain-cognition pathway in a large Chinese population and found the mediating effect of intrinsic brain activity between the association from single nucleotide polymorphism (SNP) and GMV to working memory performance. Furthermore, Gong et al. [31] utilized a brain-wide association analysis (BWAS) to investigate the associations between genetic variants and brain structure, finding positive correlations between *DISC-1* SNPs and GMV in the precuneus, post-central gyrus, and middle cingulate gyrus. The *DISC-1*-associated GMV also positively correlated with negative symptom severity [31]. In terms of functional imaging studies, genetic variants of *GRIN2A* were associated with language-related negative symptoms which correlated with FC between the left posterior superior temporal gyrus and the superior lateral occipital cortex [32]. In addition, the polygenic risk score of *MIR137* has been demonstrated negatively correlated with FC between the dorsolateral prefrontal cortex and both the superior parietal cortex and the inferior temporal cortex [33]. These findings support the potential link between genetic endophenotype and neuroimaging phenotype, which may further be involved in the pathogenesis of SCZ. Substantial neuroimaging phenotypes have been identified between DS and NDS, particularly in fMRI, but it is still unknown whether or which genes are responsible for these changes and how the genes regulate the different alterations of FC. Furthermore, it is unclear whether there are common or specific gene regulatory mechanisms between DS and NDS.

**Fig. 1:**
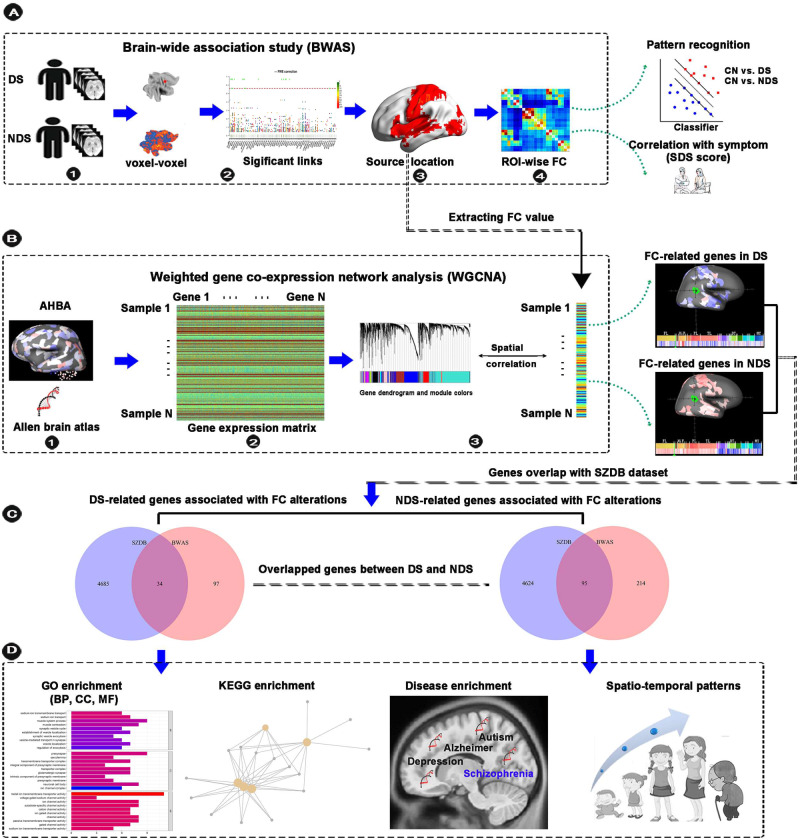
Schematic of data analysis pipeline. **A** We performed a voxel-based unbiased brain-wide association study (BWAS) method on resting-state fMRI data to identify pathology as revealed by the significantly altered functional connectivity in DS and NDS patients compared to CN after controlling for age, sex, education, intracranial volume, and framewise displacement. To further test the clinical relevance of the main identified functional links as diagnostic features of DS and NDS, we applied a pattern classification approach using the alterations in the ROI as a biomarker to test how well this could distinguish patients with DS and NDS from the CN. To further assess the clinical significance of identified altered functional links in DS and NDS, we used linear regression model to quantify the dependency between differences in functional connectivity and deficit symptom severity as assessed by the SDS scale. **B** We used a WGCNA approach to identify the transcription-neuroimaging association between BWAS FC differences in DS and NDS and gene expression from the AHBA. **C** We calculated the overlapped genes between DS-related and NDS-related genes and schizophrenia-related genes in SZDB dataset. **D** GO, KEEG, and disease enrichment analysis was applied for these overlapped and non- overlapped genes between DS and NDS. Furthermore, we showed spatio-temporal expression patterns of the identified key genes. DS deficit schizophrenia, NDS non-deficit schizophrenia, CN healthy controls, ROI region of interest, FC functional connectivity, SDS schedule for deficit syndrome, AHBA Allen Human Brain Atlas, SZDB A Database for Schizophrenia Genetic Research (http://www.szdb.org/), GO Gene Ontology, BP biological process, CC cellular component, MF molecular function, KEGG Kyoto Encyclopedia of Genes and Genomes.

To address these issues, participants recruited in the present study accepted to undergo a resting-state fMRI scan and clinical assessments. The schematic of data analysis pipeline in this study was shown in Fig. 1. Briefly, voxel-based unbiased BWAS was conducted firstly to investigate the abnormal connectivity pattern of the FC networks in DS and NDS. Then the main identified FC was used for the pattern classification of diagnosis. A linear regression model was conducted to further explore the associations between abnormal FC and symptom severity. Secondly, weighted gene co-expression network analysis (WGCNA) was performed to identify the transcription-neuroimaging association between BWAS FC differences and gene expression profiles in DS and NDS. Finally, enrichment analyses were applied to reveal the genetic profiles of DS and NDS, and the spatio-temporal expression patterns of the identified key genes were further explored. We hypothesize that DS and NDS patients show different altered patterns of FC networks in BWAS. Furthermore, convergent and divergent genes expression profiles associated with brain-wide functional connectome dysfunction are identified in DS and NDS.

## Materials and methods

### Participants

We initially recruited a total of 302 male participants, including 82 DS patients, 96 NDS patients, and 124 age-matched healthy controls (CN). All patients were enrolled at the inpatient department of the psychiatric rehabilitation unit of Yangzhou Wutaishan Hospital in Jiangsu Province, China while CN were recruited via advertisements in the local community. Of these individuals, 2 CN, 2 NDS patients, and 4 DS patients were excluded due to loss of T1 image data. 4 CN, 5 NDS patients, and 12 DS patients were excluded due to excessive motion artifacts (i.e., cumulative translation or rotation of more than 3 mm or 3°). The remaining 66 DS patients, 89 NDS patients, and 118 CN were included in the subsequent analyses (Table 1). DS and NDS patients were diagnosed according to the Chinese version of the Schedule for the Deficit Syndrome (SDS) [34]. The inclusion and exclusion criteria used to choose the subjects are presented in our previously published studies [19, 22, 35] and are provided in the SI Methods. All participants provided written informed consent, which was approved by the Institutional Ethical Committee for Clinical Research of the Affiliated Brain Hospital of Nanjing Medical University and ZhongDa Hospital Affiliated to Southeast University.

**Table 1:** Demographics, clinical measures, and head rotation parameters of NDS, DS, and CN subjects.

Items	CN	NDS	DS	*F* (T)values	*p* values
	*n* = 118	*n* = 89	*n* = 66		
Age (years)	50.76 (7.60)	49.18 (7.96)	51.20 (8.31)	1.520	0.221
Education level (years)	9.76 (2.82)	8.91 (2.47)	8.49 (3.02)^a^	5.133	0.006
CPZ-equivalent daily dose (mg/day)	–	560.94 (210.67)	532.21 (213.39)	0.8349	0.405
BPRS	–	27.26 (3.14)	31.39 (3.14)^b^	8.117	<0.001
SAPS	–	9.72 (4.54)	8.91 (3.64)	−1.193	0.235
SANS	–	33.89 (8.80)	55.58 (11.60)^b^	13.241	<0.001
SDS	–	4.81 (2.251)	12.77 (2.976)^b^	18.975	<0.001
TIV	1528.49 (117.50)	1443.71 (115.04)^a^	1471.35 (112.23)^a^	14.482	<0.001
FD	0.09 (0.04)	0.09 (0.04)	0.08 (0.05)^a^	3.525	0.031

Note: data are presented as the mean (standard deviation, SD). One-way analysis of variance (ANOVA) was used to compare differences in age, education, TIV, and FD among the three groups. Post hoc pairwise comparisons were performed by using Bonferroni comparisons. The clinical characteristics (BPRS, SANS, and SAPS) between DS and NDS groups were compared by using two independent samples *t*-test.

*CPZ* chlorpromazine, *BPRS* brief psychiatric rating scale, *SANS* assessment of negative symptoms, *SAPS* scale for the assessment of positive symptoms, *SDS* the schedule for the deficit syndrome, *TIV* total intracranial volume, *FD* framewise displacement; *NDS* non-deficit schizophrenia, *DS* deficit schizophrenia, *CN* healthy controls.

^a^Compared to CN, significant differences were found in NDS and DS.

^b^Significant differences were found between NDS and DS.

### Clinical evaluation

All patients were measured on the Brief Psychiatric Rating Scale (BPRS) [36], the Scale for the Assessment of Negative Symptoms (SANS) [37], the Scale for the Assessment of Positive Symptoms (SAPS), and SDS to assess the severity of their psychiatric symptoms.

### MRI data acquisition

All MRI data were acquired using a 3.0 Tesla magnetic resonance (MR) system (GE HDx, Chicago, Illinois) with an 8-channel phased array head coil located in the Subei Hospital of Jiangsu Province, Yangzhou, China. Resting-state functional images were acquired using a gradient recalled echo-echo planar imaging (GRE-EPI) sequence with the following parameters: repetition time (TR) = 2000 ms, echo time (TE) = 25 ms, flip angle (FA) = 90°, 35 slices, field of view (FOV) equal = 240 mm × 240 mm, slice thickness = 4 mm, gap = 0 mm, matrix size = 64 × 64, voxel size = 4 × 3.75 × 3.75 mm^3^, and 240 volumes. During the magnetic resonance imaging (MRI) scan, all participants were asked to lie awake in the scanner with their eyes closed and their head firmly positioned inside the coil to minimize head motion. The imaging process took ~8 min. High-resolution T1-weighted images were acquired by a 3D magnetization-prepared rapid gradient-echo (MPRAGE) sequence. The parameters were as follows: TR = 8.16 ms, TE = 3.18 ms, inversion time (TI) = 450 ms, slice thickness = 1.0 mm, gap = 0.5 mm, matrix = 256 × 256, FA = 12°, FOV = 128 mm × 128 mm, voxel size = 1 × 0.5 × 0.5 mm^3^.

### fMRI image preprocessing

In this study, MATLAB2016b (http://www.mathworks.com/products/matlab/) and DPABI software [38] were used to preprocess all fMRI data. The image preprocessing procedure was performed as described in our previous studies [39, 40] and is summarized in SI Methods. Briefly, the image processing procedure included slice timing correction, head motion correction, realigning, normalization, smoothing, nuisance covariate regression, and filtering.

### Brain-wide association study

#### Voxel-wise whole-brain functional connectivity analysis

The brain-wide association study could explore the differences between patients and CN in the connectivity of each pair of brain voxels at a whole-brain level, withing fully unbiased. Referring to previous studies [41], the voxel-wise whole-brain FC analysis was based on Automated Anatomical Labelling (AAL3) atlas [42]. Detailed brain regions and correspondent abbreviations in AAL3 atlas are shown in Tables S1. Every resting-state fMRI image contained 23178 voxels. First, we extracted time series from pair-wise voxels in AAL3. Secondly, Pearson cross-correlation analyses were performed in the whole-brain pair-wise voxel-level followed by Fisher’s Z-transformation. Two-tailed two-sample *t*-tests were then conducted on 268609842 (23178 × 23178)/2 z-transformed correlative coefficients for identifying significant altered functional connectivity in DS and NDS patients compared to CN. Age, education level, framewise displacement (FD), and Total Intracranial Volume (TIV) were regressed out as potential confounding factors in the analyses. To strengthen statistical power, we performed a strict Family-Wise Error (FWE) correction in multiple comparisons.

#### Calculation of a measure for the association

**Fig. 2:**
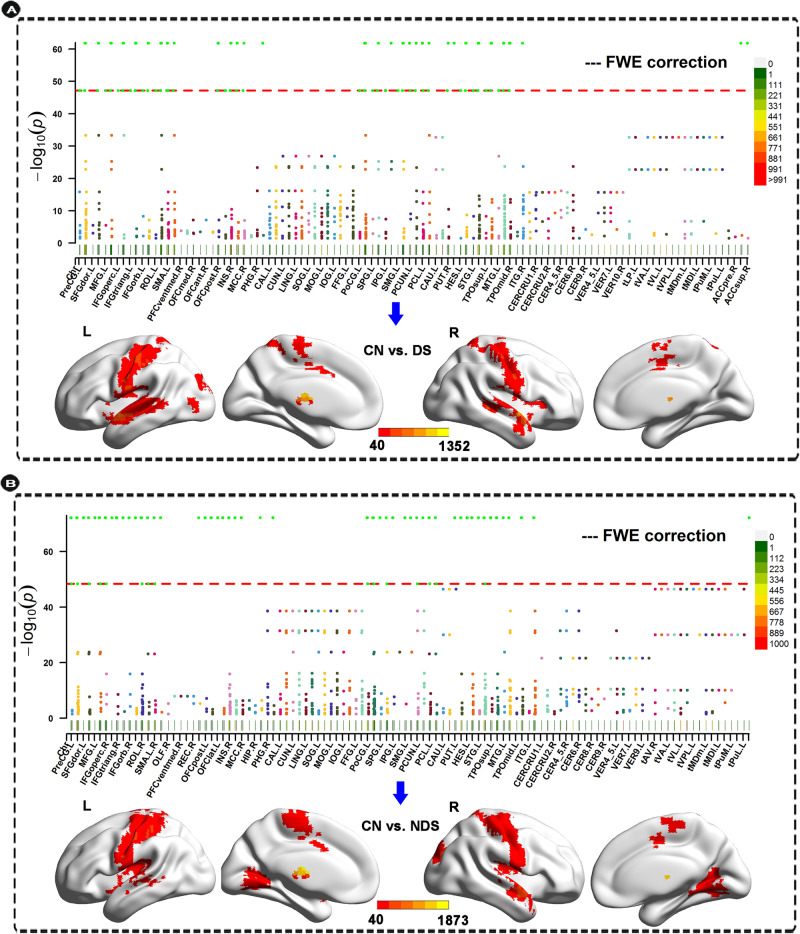
The results of voxel-based unbiased brain-wide association study in DS and NDS groups. Anatomical location of consistently altered functional connectivity in DS (**A**) and NDS (**B**) obtained from voxel-based BWAS. Manhattan plot of voxel-based unbiased brain-wide association study results indicating that DS showed significantly altered functional links compared to CN. Each dot represents a functional connectivity link between two voxels. Note there are a total of 23,178 × 23,178/2 links, and we only plot a significant dot if Cluster defining threshold (*z*-value) > 7. The red dotted line is the whole-brain FWE correction threshold *p* = 7.22 × 10^−48^. The regions indicate the AAL3 atlas regions in which the voxels were located, with the numbers for each region specified in Table S1. The glass brains indicated the anatomical location of the voxels showing significantly altered functional connectivity with other voxels (FWE < 0.05). The color bar indicates the measure of association (MA, see text) given by the number of significantly affected links relating to each voxel. We presented clusters voxels containing more than 100 significant voxels (MA > 40). DS deficit schizophrenia, NDS non-deficit schizophrenia, CN healthy controls.

A measure for the association (MA) between a voxel i and the brain disorder was defined as ${MA}(i)={{\rm{N}}}_{\alpha }$. In this formula, N_α_ represented number of links between voxel *i* and every other voxel in the entire cerebrum which shares a *p*-value < α (which in the present study with FWE correction was *p* < 0.01). A larger value of MA was considered to imply a more significant FC alteration in DS and NDS [41]. The MA value described above shows voxels with significantly different FC, but not the brain regions to which these voxels have altered connectivity. To facilitate the explanation of our results, we set two other thresholds of MA > 40 [41] and voxel cluster size >100 to show the pattern of the altered connectivity, when assessing which voxels had the significant differences between the two groups (as shown in Fig. 2).

#### Clinical significance of the altered functional connectivity

First, we made regions of interest (ROIs) based on significant clusters from the findings of BWAS. Second, we extracted average time series for all voxels within ROIs as the reference time course and performed ROI-wise cross-correlation analysis between the averaged time courses within all ROIs. It should be noted that all cluster-wise analyses are based on findings of BWAS, and that it is the BWAS statistics only on which we rely. Finally, to investigate the clinical significance of altered FC, we performed a linear regression model analysis between altered connectivity and clinical measures (SDS, BPRS, SANS, and SAPS scores). To control the false positive rate, we used a false discovery rate (FDR) procedure to correct for multiple comparisons.

#### Pattern classification based on the altered functional connectivity

To further test the clinical relevance of the main identified DS and NDS links as diagnostic features of DS and NDS, we applied two machine learning techniques (SVM, support vector machine, and LDA, linear discriminant analysis) using the FC alterations in the ROIs as a biomarker to test how well this could distinguish patients with DS and NDS from the CN. Firstly, we extracted features from the voxel-based ROI-wise altered functional connectivity from results of voxel-based BWAS (23 × 22/2 = 253 correlation coefficients for DS, 20 × 19/2 = 190 correlation coefficients for NDS). Then we used two methods (Principal Component Analysis and Recursive Feature Elimination, respectively) to reduce dimension of features. Finally, we performed SVM and LDA to distinguish patients with DS and NDS from the CN, respectively. Furthermore, we used a leave-one-out cross-validation (LOOCV) strategy to estimate the generalization of this SVM/LDA classifier and to assess its accuracy, sensitivity, and specificity. Area under the receiver operator characteristic curve (AUC) was used to indicate the classification power of this SVM/LDA classifier. Circle diagram was used to the contribution of all features in this SVM/LDA classifier for distinguishing DS and NDS from CN.

#### Gene expression data processing

The normalized gene expression data of 1280 samples (HCP parcellation) or 1281 samples (Schaefer300, Schaefer500, and Schaefer1000 parcellations) of 6 donated brains with left cortex coverage were obtained from the Allen Human Brain Atlas (AHBA, http://human.brain-map.org). A total of 58,692 probes were used to detect the gene expression in these samples [43, 44]. A newly proposed pipeline for transcription-neuroimaging association studies based on AHBA data was used in this study [45]. In all available 58692 probes, the Re-Annotator toolkit [46] was used to perform probe-to-gene reannotation with the latest information from the National Center for Biotechnology Information (NCBI). We identified 42383 probes with unique gene annotation, corresponding to 20232 unique genes. With intensity-based filtering, we excluded probes that did not exceed the background noise in at least 50% of samples across all donors. RNA-seq data were used to further select probes. After excluding genes whose expression values were not measured by the RNA-seq method, we excluded probes with low correlations (Spearman rho < 0.2) between expression values measured by microarray and RNA-seq methods. A representative probe for a gene was selected based on the highest correlation to the RNA-seq data in corresponding samples. To correct for donor specific effects, scaled robust sigmoid (SRS) normalization was used to ensure equivalent scaling of expression values for each donor. After this procedure, the expression values were more comparable across donors. Finally, 10027 candidate genes were obtained (detailed parameter selections are in Table S2). We obtained a sample × gene expression matrices of 1280 × 10027 (for HCP parcellation) or 1281 × 10027 (for Schaefer300, Schaefer500, and Schaefer1000 parcellations) from the six donors with left cortex coverage. Here, the samples represent brain tissue samples. Based on the newly proposed pipeline [45], we obtained four processed regions × gene expression matrices for the four parcellations.

#### Differential stability filtering

Differential stability score (DSS) was defined to quantify the consistency of gene expression patterns across brain regions between donors [44]. The DSS score was defined as the tendency for a gene to exhibit reproducible expression patterns across brain structures. In our study, four parcellation masks (HCP 180 regions, Schaefer 300 regions, Schaefer 500 regions, and Schaefer 1000 regions, respectively) of the cerebral cortex [45] were used to calculate DSS. The average expression value of each gene (*n* = 10,027) was calculated for each area (*n* = 180, 300, 500, and 1000 for four brain parcellations, respectively) from each donor (six left brain hemispheres). For each gene, Spearman’s correlations of gene expression across areas were performed between any two of the six donors, and the average correlation coefficient of each gene was defined as the DSS score of this gene. Genes with more consistent spatial expression patterns between donors would show a high DSS score. Since consistent gene expression patterns between individuals were prerequisites in the transcription-neuroimaging association studies, only genes (*n* = 1081, 845, 722, 628 for four brain parcellations, respectively) with DSS scores > 0.5 [44] were included in this study to ensure relatively consistent expression patterns between individuals.

#### Weighted gene co-expression network analysis

To create gene modules, we performed WGCNA, which an effective tool that clusters genes into network modules and detects biologically meaningful results [47, 48]. We first performed z-transformation for the gene expression data. Then we constructed a signed network for each sample × gene expression matrix for four brain parcellations, respectively. A WGCNA consensus network was created to identify common expression patterns across the four aforementioned expression matrices, and 3 gene modules were finally obtained (We set the threshold of module gene number to 50). For each module, the expression pattern (a matrix represented by sample × number of genes in this module) was summarized by the first principal component, defined as the module eigengene (ME, number of samples × 1 vector).

#### Transcription-neuroimaging association analysis

To establish the stable sample correspondence between gene expression and neuroimaging data, we defined three radius spheres (4 mm, 6 mm, and 8 mm) centered at the MNI coordinate of each tissue sample and extracted the mean value on each aforementioned MA-value map of FC differences. Then, for each module, we calculated Pearson’s correlation between the ME and the MA-value across brain tissue samples. Multiple comparisons were corrected using the FDR method (*q* < 0.05). To control the effect of radius spheres, the significant gene modules must be significant at all three radius spheres. Based on the significant positive and negative correlations between the ME and the MA-value in brain tissue samples, we define genes in modules as the positive and negative correlation genes. Then based on the results of correlation analyses, we first intersected the positive and negative correlation genes of 4 brain parcellations. Furthermore, to make sure the genes were from brain tissue and were associated with SCZ, we calculated the genes that intersected the SCZ genes expressed in brain tissue in the A Database for Schizophrenia Genetic Research (SZDB) (http://www.szdb.org/) [49]. We obtained DS-related genes associated with FC alterations and NDS-related genes associated with FC alterations. Finally, we calculate the overlapped positive and negative regulation genes between DS and NDS. Furthermore, to identify key genes associated DS and NDS, we also calculated Pearson’s correlation between the positive and negative regulation gene expressions of overlapped genes and non-overlapped genes, respectively. We defined the genes with correlation values greater than 0.7 as identified key genes.

#### Enrichment analysis

Enrichment analysis for identified key genes of overlapped genes between DS and NDS was mainly performed by the clusterProfiler4.0: R software package (https://bioconductor.org/packages/clusterProfiler/), including the Gene Ontology (GO), Kyoto Encyclopedia of Genes and Genomes (KEGG), and Disease Ontology analysis (DO) databases. We also showed these key genes expression differences in SCZ patients compared with healthy controls from SZDB (http:// www.szdb.org/) when genes were enriched in function, pathway or disease.

#### Spatio-temporal expression patterns

To investigate the spatio-temporal expression patterns in human brain of the identified key genes [50], we extracted spatio-temporal expression patterns of the identified key genes from Human Brain Transcriptome web (https://hbatlas.org/). We showed dynamic gene expression along entire development and adulthood in the 11 areas of neocortex (NCX). To investigate the spatio-temporal dynamics of the human brain transcriptome, we created a 15-period system spanning the periods from embryonic development to late adulthood (Table S3).

## Results

### Demographic and clinical features

The demographic data for all participants is presented in Table 1. There were significant differences found for education, TIV and FD (all *p* < 0.05) among groups, but not in age. There were no significant differences for antipsychotic medicine dosage between the two patient subgroups. For the clinical features, there were significant differences in scores of BPRS, SANS and SDS between patients with DS and NDS (all *p* < 0.001). Additionally, there was no significant difference in scores of SAPS between patient groups (*p* > 0.05).

### Whole-brain voxel-based functional networks

As shown in Fig. 2, both DS and NDS showed a number of voxel clusters with different FC compared to CN. These voxels had some FC that were significantly different across the whole brain after FWE correction, with the FWEp < 0.05, the significance level uncorrected had to be *p* = 7.22 × 10^−48^. In addition, comparison between DS and NDS also showed significant differences in FC of the whole brain after FWE correction (Fig. S1).

### Altered functional connectivity pattern

To investigate the abnormal connectivity pattern in the FC networks in DS and NDS, all significant voxels (after FWE correction) were parcellated into regions according to the AAL3 atlas [42]. On this basis, we identified 23 ROIs in DS and 20 ROIs in NDS. Figure 2 and Table 2 showed the FC differences between the significantly different voxels in these regions (MA > 40, significant voxels >100). These regions in DS are mainly located in bilateral postcentral gyrus, bilateral precentral gyrus, bilateral middle temporal gyrus, bilateral superior temporal gyrus, bilateral middle occipital gyrus, right supplementary motor area, bilateral calcarine fissure & surrounding cortex, bilateral lingual gyrus, bilateral insula, right rolandic operculum, right middle cingulate gyrus, right fusiform gyrus, right superior occipital gyrus, left superior parietal gyrus, and left paracentral lobule. These regions in NDS are mainly located in bilateral postcentral gyrus, bilateral precentral gyrus, bilateral superior temporal gyrus, bilateral calcarine fissure & surrounding cortex, bilateral middle temporal gyrus, bilateral lingual gyrus, right supplementary motor area, bilateral insula, left middle occipital gyrus, right fusiform gyrus, right rolandic operculum, left paracentral lobule, and right middle occipital gyrus.

**Table 2:** Significant ROIs in the voxel-based BWAS analysis in DS and NDS compared to CN.

No.	Regions	Voxels in ROI	Peak MA value	MNI (peak)
CN vs. DS				
ROI1	Left postcentral gyrus	400	494	−50, −14, 48
ROI2	Right postcentral gyrus	360	398	42, −22, 36
ROI3	Right precentral gyrus	297	466	50, −14, 56
ROI4	Left middle temporal gyrus	267	335	−66, −38, 4
ROI5	Right superior temporal gyrus	256	1352	50, −2, −4
ROI6	Left precentral gyrus	223	490	−42, −18, 64
ROI7	Left superior temporal gyrus	213	1115	−50, −2, −4
ROI8	Left middle occipital gyrus	209	224	−18, −90, 16
ROI9	Right supplementary motor area	188	226	6, −6, 48
ROI10	Right middle temporal gyrus	174	320	46, −62, 4
ROI11	Left calcarine fissure & surrounding cortex	140	113	−2, −98, 12
ROI12	Right lingual gyrus	140	76	26, −62, 0
ROI13	Right insula	134	1126	50, 2, −4
ROI14	Right rolandic operculum	134	419	50, −2, 4
ROI15	Left insula	134	306	−46, −6, 0
ROI16	Right calcarine fissure & surrounding cortex	134	61	18, −58, 12
ROI17	Right middle cingulate gyrus	133	272	6, −6, 44
ROI18	Left lingual gyrus	123	85	−14, −62, −4
ROI19	Right fusiform gyrus	119	85	30, −54, −4
ROI20	Right middle occipital gyrus	113	194	26, −94, 8
ROI21	Right superior occipital gyrus	110	214	22, −94, 8
ROI22	Left superior parietal gyrus	103	242	−18, −50, 64
ROI23	Left paracentral lobule	103	93	−6, −18, 68
CN vs. NDS				
ROI1	Left postcentral gyrus	410	626	−38, −26, 40
ROI2	Right postcentral gyrus	352	496	30, −30, 52
ROI3	Right precentral gyrus	312	414	34, −26, 52
ROI4	Left precentral gyrus	250	292	−58, −6, 32
ROI5	Right superior temporal gyrus	231	628	54, −2, −8
ROI6	Left superior temporal gyrus	205	534	−50, −6, −4
ROI7	Right calcarine fissure & surrounding cortex	186	238	26, −70, 12
ROI8	Left middle temporal gyrus	177	124	−66, −38, 0
ROI9	Right lingual gyrus	175	285	22, −50, 0
ROI10	Right supplementary motor area	171	237	2, −6, 48
ROI11	Left lingual gyrus	165	293	−18, −66, −4
ROI12	Left insula	162	260	−38, 22, 0
ROI13	Left calcarine fissure & surrounding cortex	154	289	−18, −74, 4
ROI14	Right insula	150	352	50, −2, 0
ROI15	Left middle occipital gyrus	142	231	−22, −82, 20
ROI16	Right fusiform gyrus	142	161	42, −34, −28
ROI17	Right middle temporal gyrus	140	405	54, −2, −16
ROI18	Right rolandic operculum	136	299	50, −6, 8
ROI19	Left paracentral lobule	126	276	−10, −18, 64
ROI20	Right piddle occipital gyrus	100	438	26, −82, 16

*DS* deficit schizophrenia, *NDS* non-deficit schizophrenia, *CN* healthy controls, *ROI* region of interest, *FC* functional connectivity, *BWAS* brain-wide association study.

### Clinical significance of the altered functional connectivity

**Fig. 3:**
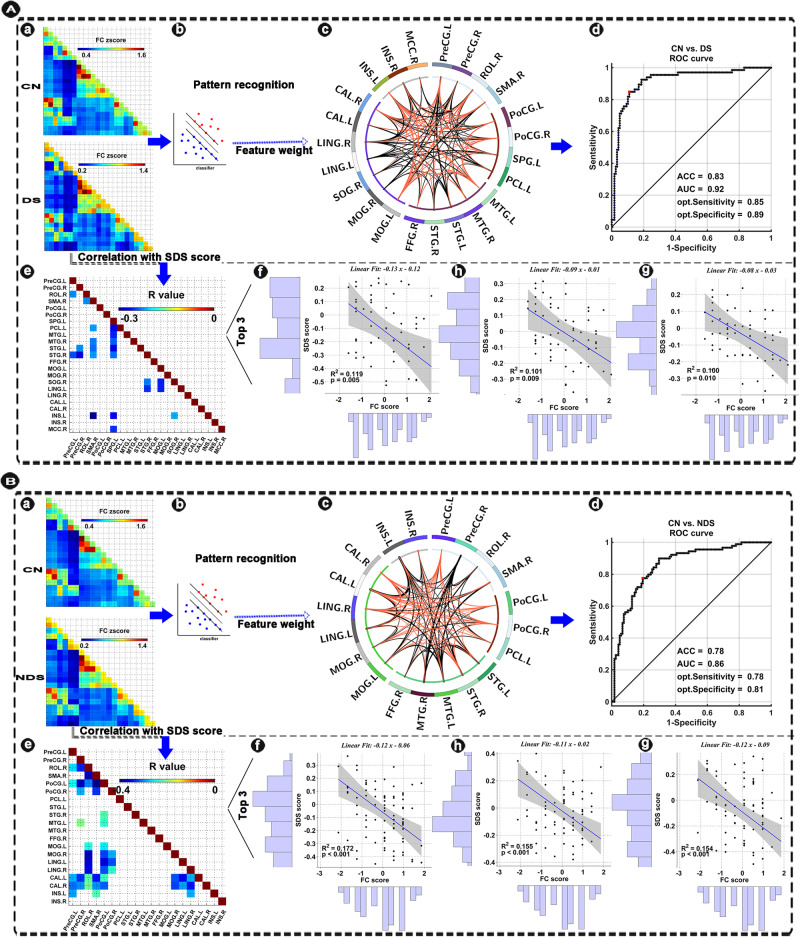
Pattern classification power of identified altered functional links in DS and NDS from CN and its clinical significance. **A**-a, **B**-a Functional connectivity matrix (i.e., features) were extracted from the ROI-wise functional connectivity (23 × 22/2 = 253 correlation coefficients for DS vs. CN; 20 × 19/2 = 190 correlation coefficients for NDS vs. CN) from results of voxel-based BWAS. **A**-b, **B**-b Indicating the schematic diagram of pattern recognition. **A**-c, **B**-c Circle diagram showed the contribution of all features in MRI-based “classifier” for distinguishing DS and NDS from CN. Red links indicate negative weight and black links Indicate positive weight. The thickness of links indicates the weight value. **A**-d, **B**-d ROC curve shows the classification power in MRI-based “classifier” for discriminating DS and NDS from CN. **A**-e, **B**-e Indicating the significant correlations between FC differences and deficit symptom severity in DS. **A**-f, h, g; **B**-f, h, g Indicating relationships between FC differences and SDS score (i.e. deficit symptom severity) in DS in the top three significant correlations. DS deficit schizophrenia, NDS non-deficit schizophrenia, CN healthy controls, FC functional connectivity, BWAS brain-wide association study, ACC accuracy, AUC area under the ROC curve, Opt optimum, ROC receiver operating characteristic, SDS Schedule for deficit syndrome. The anatomical abbreviations are for the areas in the AAL3 atlas, with abbreviations shown in Table S1.

As shown in Fig. 3, the linear regression model analysis showed that the FC changes between the significant voxels in the 23 ROIs AAL3-based areas of DS and 20 ROIs of NDS were significantly negatively correlated with the SDS scores.

### Classification of DS and NDS based on the altered functional connectivity links

To further test the functional significance of the links identified as related to DS and NDS, we used two machine learning techniques (SVM and LDA) to investigate the utility of the altered network as a biomarker for distinguishing DS and NDS from CN. The LDA classifier’s classification accuracy was 83% for distinguishing DS from CN. As shown in Fig. 3A-d, the LDA classifier’s ROC curve shows a high power to distinguish DS patients from CN on an individual subject basis, with an AUC of 92%, 85% sensitivity, and 89% specificity. The LDA classifier’s classification accuracy was 78% for distinguishing NDS from CN. As shown in Fig. 3B-d, the LDA classifier’s ROC curve shows a high power to distinguish NDS patients from CN on an individual subject basis, with an AUC of 86%, 78% sensitivity, and 81% specificity. The LDA classifier’s ROC curve shows an AUC of 66%, 62% sensitivity, and 74% specificity in distinguishing DS from NDS patients (Fig. S2). Furthermore, the results of SVM are provided in Fig. S3 and SI Results because the identification ability of LDA is better than that of SVM.

### Transcription-neuroimaging stable associations

**Fig. 4:**
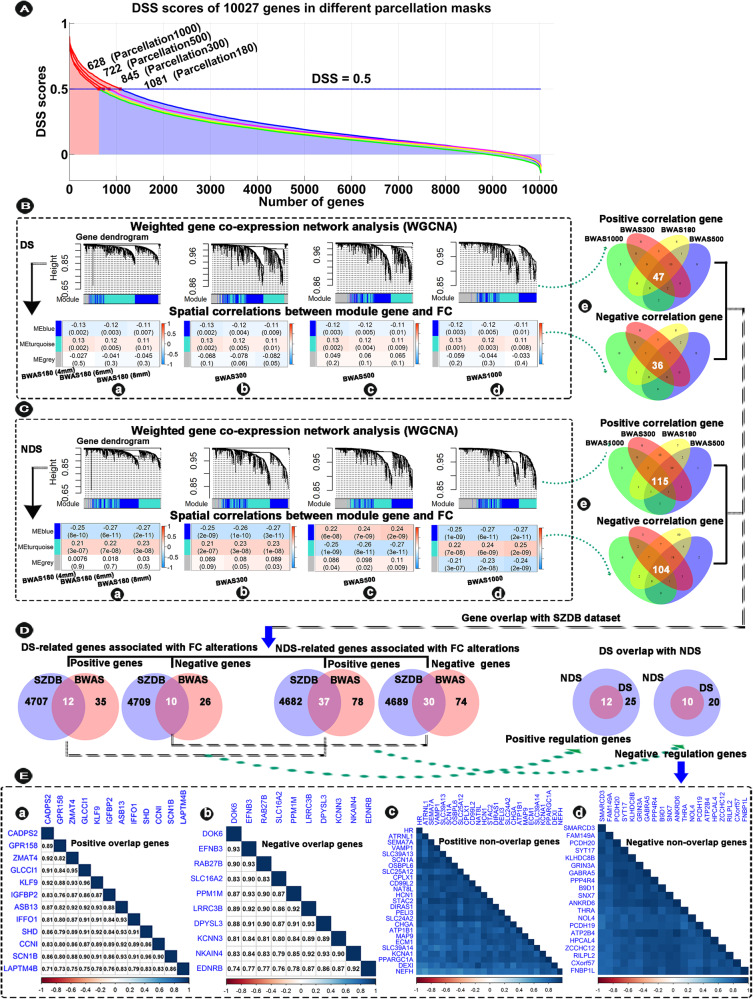
Transcription-neuroimaging association between BWAS FC differences in DS and NDS and gene expression from the AHBA (six left hemispheres). **A** The distribution of the DSS scores of the 10027 genes in different parcellation masks: parcellation 1000 (500 regions for each hemisphere), parcellation 500 (250 regions for each hemisphere), parcellation 300 (150 regions for each hemisphere), and parcellation 180 (HCP 180 regions for each hemisphere), respectively. **B**, **C** WGCNA dendrogram showing consensus modules based on the topological co-expression of genes with DSS >0.5. The heatmap shows spatial correlations (positive and negative correlations) between MEs of gene modules and FC alterations in parcellation 180 (BWAS180), parcellation 300 (BWAS300), parcellation 500 (BWAS500), parcellation 1000 (BWAS1000) in DS and NDS. Venn diagram shows gene numbers derived from different parcellation masks and their overlaps. Different colors indicate the numbers of genes derived from different parcellation masks. Note, we show positive and negative correlations, separately. **D** Venn diagram shows the overlaps between DS-related and NDS-related genes and schizophrenia-related genes in SZDB dataset, and shows gene numbers derived from DS and NDS and their overlaps. **E** The heatmap shows correlation matrix of gene expression of overlapping genes (**E**-a, b) and non-overlapped genes (**E**-c, d) between DS and NDS. Note, the full names of all gene abbreviations are shown in Table S4. DS deficit schizophrenia, NDS non-deficit schizophrenia, DSS differential stability score, WGCNA weighted gene co-expression network analysis, AHBA Allen Human Brain Atlas, SZDB A Database for Schizophrenia Genetic Research (http://www.szdb.org/).

Three consensus modules were identified from WGCNA analysis based on expression data from six left-hemispheric brain samples (Fig. 4B, C). Cross-sample Pearson’s correlation was calculated between MEs of each module and FC differences to show the consistent correlation pattern of modules across the four parcellations and the three radius spheres of each tissue sample. The results showed that there were 47 consistently positive DS-related genes and 36 negative DS-related genes in significant genes module (overlapping four parcellations) (Fig. 4a–e), and 115 consistently positive NDS-related genes and 104 negative DS-related genes in significant genes module (overlapping four parcellations) (Fig. 4a–e). The positive correlation means that brain regions with significant FC reduction in DS and NDS show higher gene expression, and the negative correlation means that those regions show lower gene expression.

Furthermore, to test whether these identified genes are related to brain expression genes of patients with SCZ, we calculated overlap of these identified genes and schizophrenia-related genes in SZDB dataset. We obtained 12 consistently positive DS-related genes and 10 negative DS-related genes, and 37 consistently positive NDS-related genes and 30 negative NDS-related genes (Fig. 4D). Finally, we found 12 positive and 10 negative regulation overlap genes between NDS and DS (Fig. 4D and the full names of genes are provided in Table S4) and there is a high correlation between these genes (Fig. 4E).

To avoid the scenario that co-expression networks constructed accidentally based on the set of WGCNA parameters used, we rebuilt networks by applying different parameter sets. The final consensus modules used in this study were conserved among all the tests with different parameter sets (Fig. S4).

### Enrichment analysis

**Fig. 5:**
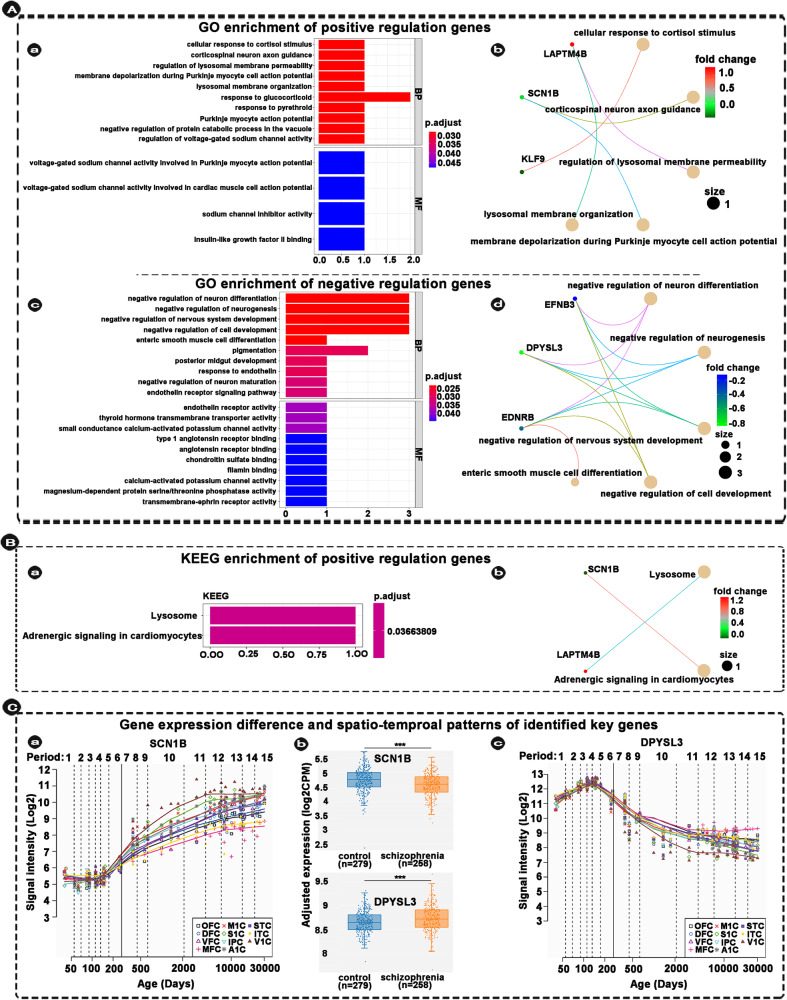
Enrichment of overlapped genes between DS and NDS and spatio-temproal patterns of identified key genes. **A** Indicating GO enrichment of positive and negative overlapped genes between DS and NDS (a, c), and indicating the relationships between identified genes and top five significant pathways (b, d). **B** Indicating KEEG enrichment and the relationships between identified genes and significant pathways. **C** Indicating genes expression differences and spatio-temporal expression patterns of the identified key genes. Each changing curve represents the change in gene expression throughout the lifespan. The box plot represents differences in gene expression of these two key genes in schizophrenia patients compared with healthy controls (Note: the box plot is from SZDB, http://www.szdb.org/). *p* < 0.001. DS deficit schizophrenia, NDS non-deficit schizophrenia, DSS differential stability score, WGCNA weighted gene co-expression network analysis, AHBA Allen Human Brain Atlas, SZDB A Database for Schizophrenia Genetic Research (http://www.szdb.org/), BP biological processes, CC cellular components, MF molecular function, OFC orbital prefrontal cortex, DFC dorsolateral prefrontal cortex, VFC ventrolateral prefrontal cortex, MFC medial prefrontal cortex, M1C primary motor (M1) cortex, S1C primary somatosensory (S1) cortex, IPC posterior inferior parietal cortex, A1C primary auditory (A1) cortex, STC superior temporal cortex, ITC inferior temporal cortex, V1C primary visual (V1) cortex. Note: all results show only results of significant enrichment.

**Fig. 6:**
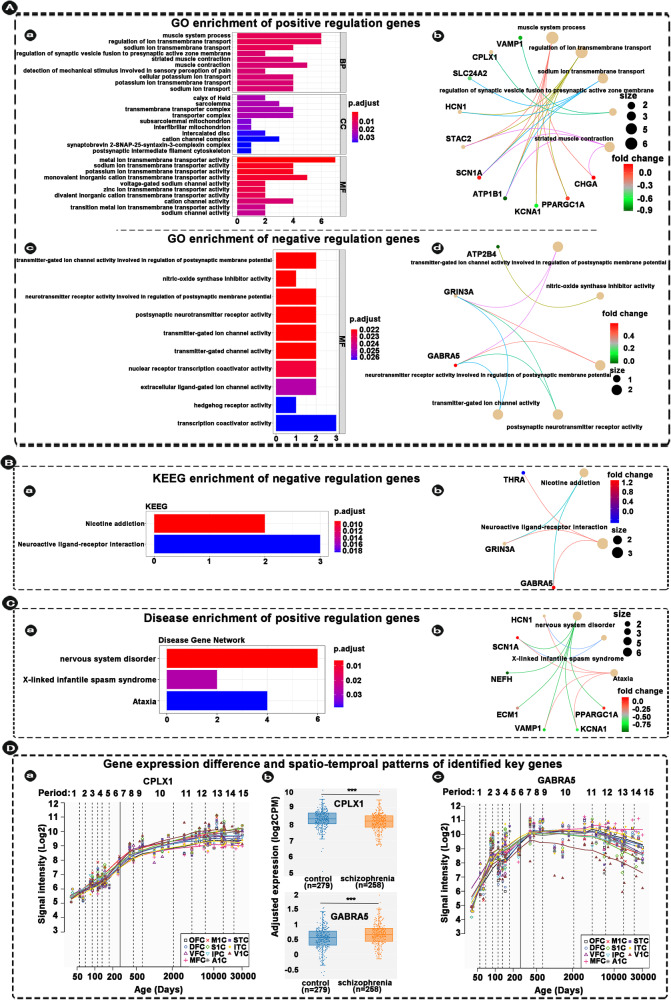
Enrichment of non-overlapped genes between DS and NDS and spatio-temproal patterns of identified key genes. **A**-a, c Indicating GO enrichment of positive and negative non-overlapped genes between DS and NDS. **A**-b, d Indicating the relationships between identified genes and top five significant pathways. **B** Indicating KEEG enrichment and the relationships between identified genes and significant pathways. **C** Indicating disease-related genes enrichment and the relationships between identified genes and significant pathways. **D** Indicating genes expression differences and spatio-temporal expression patterns of the identified key genes. Each changing curve represents the change in gene expression throughout the lifespan. The box plot represents differences in gene expression of these two key genes in schizophrenia patients compared with healthy controls (note: the box plot is from SZDB, http://www.szdb.org/). *p* < 0.001. DS deficit schizophrenia, NDS non-deficit schizophrenia, DSS differential stability score, WGCNA weighted gene co-expression network analysis, AHBA Allen Human Brain Atlas, SZDB A Database for Schizophrenia Genetic Research (http://www.szdb.org/), OFC orbital prefrontal cortex, DFC dorsolateral prefrontal cortex, VFC ventrolateral prefrontal cortex, MFC medial prefrontal cortex, M1C primary motor (M1) cortex, S1C primary somatosensory (S1) cortex, IPC posterior inferior parietal cortex, A1C primary auditory (A1) cortex, STC superior temporal cortex, ITC inferior temporal cortex, V1C primary visual (V1) cortex. Note: all results show only results of significant enrichment.

GO enrichment analysis was conducted for positive and negative regulation overlapped/non-overlapped genes between NDS and DS, respectively (Figs. 5 and 6). None of enrichment items for positive and negative overlapped genes can be obtained in cellular components (FDR-BH *p* < 0.05, *q* < 0.2).

As shown in Fig. 5A-a,b, the top 10 pathways in which positive regulation overlap genes are enriched in biological processes are associated with cellular response to cortisol stimulus (*KLF9*), corticospinal neuron axon guidance (*SCN1B*), regulation of lysosomal membrane permeability (*LAPTM4B*), membrane depolarization during Purkinje myocyte cell action potential (*SCN1B*), lysosomal membrane organization (*LAPTM4B*), response to glucocorticoid (*IGFBP2*/*KLF9*), response to pyrethroid (*SCN1B*), Purkinje myocyte action potential (*SCN1B*), negative regulation of protein catabolic process in the vacuole (*LAPTM4B*), and regulation of voltage-gated sodium channel activity (*SCN1B*). The 4 pathways in which positive regulation overlap genes are enriched in molecular function are associated with voltage-gated sodium channel activity involved in Purkinje myocyte action potential (*SCN1B*), voltage-gated sodium channel activity involved in cardiac muscle cell action potential (*SCN1B*), sodium channel inhibitor activity (*SCN1B*), and insulin-like growth factor II binding (*IGFBP2*).

As shown in Fig. 5A-c,d, the top 10 pathways in which negative regulation overlap genes are enriched in biological processes are associated with negative regulation of neuron differentiation (*EDNRB*/*DPYSL3*/*EFNB3*), negative regulation of neurogenesis (*EDNRB*/*DPYSL3*/*EFNB3*), negative regulation of nervous system development (*EDNRB*/*DPYSL3*/*EFNB3*), negative regulation of cell development (*EDNRB*/*DPYSL3*/*EFNB3*), enteric smooth muscle cell differentiation (*EDNRB*), pigmentation (*EDNRB*/*RAB27B*), posterior midgut development (*EDNRB*), response to endothelin (*EDNRB*), negative regulation of neuron maturation (*EDNRB*), and endothelin receptor signaling pathway (*EDNRB*). The top 10 pathways in which negative regulation overlap genes are enriched in molecular function are associated with endothelin receptor activity (*EDNRB*). thyroid hormone transmembrane transporter activity (*SLC16A2*), small conductance calcium-activated potassium channel activity (*KCNN3*), type 1 angiotensin receptor binding (*EDNRB*), angiotensin receptor binding (*EDNRB*), chondroitin sulfate binding, filamin binding (*DPYSL3*), calcium-activated potassium channel activity (*DPYSL3*), magnesium-dependent protein serine (*KCNN3*), threonine phosphatase activity (*PPM1M*), and transmembrane-ephrin receptor activity (*EFNB3*).

KEGG enrichment analysis revealed that positive regulation overlap genes were related to Lysosome and Adrenergic signaling in cardiomyocytes (Fig. 5B).

None of disease enrichment items for positive and negative overlapped genes can be obtained.

### Temporal expression patterns of the identified overlapped/non-overlapped genes between NDS and DS

We extracted the lifespan expression changes of genes of interest from the Human Brain Transcriptome (HBT; https://hbatlas.org/). We categorized the spatial and temporal expression profiles in the neocortex of the identified key genes into 11 patterns. Representative identified key genes of these patterns are shown in Figs. 5C and 6C (It is note that we only present the spatio-temporal patterns of two identified key genes in the main text, respectively. Other non-representative identified key genes are shown in Fig. S5). In identified overlapped genes, the *SCN1B* gene, which is less expressed in people with SCZ than in the healthy controls (Fig. 5C-b), shows the lowest expression at the initiation of embryonic development and gradually increases after fifth period (early mid-fetal, Fig. 5C-a and Table S3). The *DPYSL3* gene, which is more expressed in people with SCZ than in the healthy controls (Fig. 5C-b), shows rapidly increased in the fetal and gradually decreases after birth, and the highest expression at the fifth period of human development (Fig. 5C-a and Table S3).

## Discussion

In the present study, we primarily demonstrated the abnormal voxel-based FC between DS, NDS, and CN, as well as the associations between resting brain FC and gene expression in DS and NDS patients. Specifically, the main findings include (1) different brain regions with altered FC were identified in DS and NDS, and the FC among regions performed a high power in classification of diseases diagnosis; (2) significant positive and negative correlations between DS/NDS-related genes and altered FC were found, and 22 overlap genes, including 12 positive and 10 negative regulation genes, between NDS and DS were further obtained; (3) the relationships between identified overlapped/non-overlapped genes and significant pathways were revealed by enrichment analysis; and (4) the specific spatial and temporal expression profiles of overlapped/non-overlapped key genes of DS and NDS were indicated. To the best of our knowledge, this is the first study that demonstrates the genes expression profiles associated with brain-wide FC dysfunction in patients with DS and NDS.

The present studies have demonstrated the differential FC in brain regions of patients with DS and NDS compared with CN. Specifically, both DS and NDS patients showed abnormal voxel-wise FC in widespread regions, including pre/postcentral gyrus, temporal gyrus, occipital gyrus, calcarine fissure, lingual gyrus and insula. Previous studies have reported inconsistent changes of FC in these brain regions, but most of them have shown relationships with clinical symptoms and cognitive changes [51–53]. The extensive FC abnormalities also support that SCZ is one of the disconnection disorders [54–56]. Additionally, our previous neuroimaging studies have shown altered neural activity and FC in the calcarine fissure, lingual gyrus and insula in patients with DS [20, 21], and the present study further validates these findings.

Moreover, the present study demonstrated that the FC values of differential brain regions showed good performances in classification of machine learning models, suggesting the potential value of functional neuroimaging features in the diagnosis of DS. Many previous studies included the FC values into the model of diagnosis classification and got better results [57, 58]. However, most of these studies identified differential brain regions based on the results of ALFF or ReHo, or based on a priori hypothesis. In contrast, our study screened out the discrepant brain regions based on data-driven analysis of BWAS, and subsequently constructed FC networks, which may provide better robustness and repeatability. In the current study, significant correlations were also observed between abnormal FC and deficit syndrome. Based on the double validation from machine learning and the relationships with deficit syndrome, these brain regions exhibiting abnormal FC may serve as potential imaging biomarkers for DS.

The method of WGCNA using in the present study, was previously reported can be used to describe the correlation structure between gene expression profiles and image data [59]. However, most previous study highlighted the relationships between brain gene co-expression networks, clinical state, peripheral blood immune indexes, polygenic risk, etc., to identify the potential biomarkers for SCZ [60–63]. In the present study, based on AHBA data and verified by different setting parameters, we obtained three gene-neuroimaging co-expression modules in both DS and NDS groups, namely positive correlation, negative correlation and none correlation, suggesting the functional changes of brain regions may related by the expression of risk genes of SCZ. The AHBA using here could improve the understanding of spatial variations on molecular scale relate to the macroscopic neuroimaging phenotypes [45]. In the present study, we mainly focused on the SCZ-related genes, especially the 22 overlapped and 45 non-overlapped genes between DS and NDS. Furthermore, the associated genes identified in our results were similarly reported in previous studies [49]. It is worth noting that all the positive and negative regulation genes of the DS group are included in the corresponding regulation genes of the NDS group, suggesting that DS has a more specific and more limited gene-image association pattern compared with NDS, which also indicated that DS is one of the special subtypes of SCZ.

In this study, the identified FC-related genes were enriched for various biological processes and molecular functions, including the significant pathways of cellular response, neuro regulation, receptor binding, etc. Among these, *SCN1B*, as one of the hub genes, was detected in the overlap positive regulation genes and was related to regulation of functions of sodium channel activity. *SCN1B* is mainly involved in the pathogenesis of epilepsy, and no previous study had reported the association between *SCN1B* and schizophrenia for far. However, *SCN2A*, also a gene of sodium channel regulator, has been reported a strong association with SCZ [64]. From another perspective, the result of *SCN1B* in the present study may provide a novel insight for subsequent studies. In addition, the *DPYSL3* was detected in the overlap negative regulation genes and was involved in negative regulation of nervous functions. Previous study had reported that *DPYSL3* was a key gene and its altered expression was associated with the neurodevelopmental and neuro-morphological pathologies present in SCZ [65]. Furthermore, the hub genes of *CPLX1* and *GABRA5* found in non-overlapped genes also indicated the complex genetic regulations in the formation, development, and plasticity of brain FC. We further investigated the spatio-temporal patterns of hub genes, and revealed the alteration of gene enrichment from embryonic to late adulthood. Previous study also explored the 349 genes in 108 schizophrenia-associated loci, revealed that cortex-specific genes were particularly expressed in the fetal brain and adult neocortex [66]. These findings demonstrated the potential neuro-imaging mechanisms underlying the pathogenesis of SCZ and need to be further verified.

Several limitations should be mentioned in the present study. First, all the SCZ patients enrolled in this study were male. The main purpose was to increase the homogeneity of patients and to better investigate the pathogenesis of SCZ. In spite of this, future studies should consider to recruit female participants in a large dataset to explore the important gender differences and to improve the statistical power of the study. Secondly, patients were treated with long-term antipsychotics, which may potentially affect brain FC and neural activity. In the present study, the types and dosages of antipsychotics were suitably matched. However, the potential impact of antipsychotics should be considered and exclude. As an improvement, first-episode drug-naive patients with SCZ could be collected for subsequent studies and analyses. Finally, this study should be considered as an exploratory analysis with a small sample size and required further verification of the results. Subsequent studies could expand the sample size and perform stricter statistical correction to explore the genetic-imaging pathogenesis of SCZ in a larger scale and multi-dimensional perspective.

In summary, the present study demonstrated different altered patterns of FC in DS and NDS patients. The overlap and non-overlap FC-related genes between two patient groups indicated the distinctive genetic-imaging pathogenesis of DS. Furthermore, the associations between gene expression and neuroimaging revealed the specific and common genetic regulation underlying these brain functional changes in DS and NDS, suggesting a novel genetic-imaging perspective for the intervention of schizophrenia.

### Supplementary information

Supplementary Materials

## Data Availability

The data that support the findings of this study are available from the corresponding authors, XRZ and JC, upon reasonable request.
