# Supplementary material for: Convergent and divergent genes expression profiles associated with brain-wide functional connectome dysfunction in deficit and non-deficit schizophrenia
Source: Transl Psychiatry. 2024 Feb 27;14:124. doi: 10.1038/s41398-024-02827-w (PMC10899251; doi:10.1038/s41398-024-02827-w)
Supplement: Supplementary file 1 — Supplementary Materials [file 41398_2024_2827_MOESM1_ESM.docx]

**Convergent and divergent** **genes expression profiles associated with brain-wide functional connectome dysfunction in deficit and non-deficit schizophrenia**

**Supplementary Materials**

**Methods**

**Inclusion and exclusion criteria**

The eligibility criteria for patients included: (1) conforming to the Diagnostic and Statistical Manual of Mental Disorders (DSM)-IV diagnosis of SCZ, which was confirmed by the Chinese version of the Structured Clinical Interview for DSM-IV (SCID-I) (First, Spitzer, Gibbon, & Williams, 2002); (2) right-handed Han Chinese patients between the ages of 20 and 65 years; and (3) showing stable psychiatric symptoms and taking antipsychotic medications for at least the 12 months prior to participation. In addition, patients were excluded if severe comorbid conditions (i.e., neurological disorders) existed, such as head trauma, mental retardation, alcoholism or substance abuse disorder, or a history of previous electroconvulsive therapy. DS and NDS patients were diagnosed according to the Chinese version of the Schedule for the Deficit Syndrome (SDS) (Wang, Yao, Kirkpatrick, Shi, & Yi, 2008). Importantly, the SDS assesses the deficit syndrome presented in patients if they have two of the following symptoms: restricted affect, diminished emotional range, poverty of speech, curbing of interests, diminished sense of purpose, and diminished social drive, the latter had to be at least moderately severe, persistent over 12 months, and not caused by secondary source such as medication side effects, depression, paranoia, or anxiety. The 124 male CN were assessed via unstructured clinical interviews in order to exclude: (1) a history of organic brain disorders, intellectual disability, or severe head trauma; (2) a history of neurological or psychiatric illnesses; (3) a family history of psychiatric disorders in first degree relatives; (4) any serious physical disease; and (5) contraindications for MRI. Sample-size were confirmed by an online sample-size calculation tool (<http://powerandsamplesize.com/>) with α=5%, 1-β=0.9, patients : CN =1 : 1.

**fMRI image preprocessing**

All fMRI data were preprocessed using MATLAB2016b (http://www.mathworks.com/products/matlab/) and DPABI image processing software (Yan, Wang, Zuo, & Zang, 2016). The image processing procedure was as previously described (Chen et al., 2022; Yan, Craddock, Zuo, Zang, & Milham, 2013). The image processing procedure was as follows: the first ten volumes were discarded to reduce the instability of MRI signal. Corrections were performed for the intra-volume acquisition time differences among slices and inter-volume motion effects during the scan (slice timing correction and head motion correction) (Power, Barnes, Snyder, Schlaggar, & Petersen, 2012; Van Dijk, Sabuncu, & Buckner, 2012). Participants with excessive head motion (cumulative translation or rotation > 3.0 mm or 3.0°) were excluded. Individual functional and structural images were co-registered. We used the Diffeomorphic Anatomical Registration Through Exponentiated Lie Algebra (DARTEL) algorithm to normalize and segment the structural images into GM, WM and cerebrospinal fluid (CSF) partitions, containing the native and DARTEL versions (Ashburner & Friston, 2009). Total intracranial volumes (TIV) were computed based on native GM, WM, and CSF by using in-home MATLAB codes (Chen et al., 2022; Chen et al., 2020). After realigning, functional images were normalized by DARTEL into MNI space (resampling voxel size, 4 × 4 × 4 mm³) and then spatially smoothed by a Gaussian kernel of 8 mm3 full-width at half maximum (FWHM) to reduce spatial noise. We next used a Friston 24-parameter model (i.e., 6 head motion parameters, 6 head motion parameters one time point before, and the 12 corresponding squared items) to regress out head motion effects from the realigned data (Friston, Williams, Howard, Frackowiak, & Turner, 1996). The WM, CSF as well as the linear trend were regressed as nuisance covariates. After realigning, slice timing correction, and co-registration, framewise displacement (FD) was calculated for all resting state volumes (Power et al., 2012). All volumes with a FD greater than 0.2 mm were regressed out as nuisance covariates (Brady et al., 2019). Any scan with 50% of volumes removed was discarded (Brady et al., 2019). Temporal band-pass filtering (0.01–0.1 Hz) was applied to reduce the effect of low-frequency drifts and high-frequency physiological noise (Chen et al., 2022; Yan et al., 2013; Zhou et al., 2019).

**Voxel-wise whole-brain functional connectivity (FC) analysis**

Detailed brain regions and correspondent abbreviations in AAL3 atlas are shown in ***Tables S1***.

**Gene expression data processing**

Detailed parameter selections in gene expression data processing are shown in ***Table S2.***

**Spatio-temporal expression patterns**

The specific 15 periods spanning the periods from embryonic development to late adulthood are shown in ***Table S3*.**

**Results**

**Whole-brain voxel-based functional networks**

The results of significant different FC between DS and NDS are showed in ***Figure S1***.

**Classification of** **DS and NDS based on the altered functional connectivity links**

The result of LDA classification for distinguishing DS from NDS is provided in ***Figure S2***. The results of SVM are provided in ***Figure S3.***

**Transcription-neuroimaging stable associations**

The full names of overlap genes identified between DS and NDS are provided in ***Table S4.*** The results of WGCNA with different parameter sets are shown in ***Figure S4***.

**Temporal Expression Patterns of the Identified Overlapped/Non-overlapped Genes between NDS and DS**

The temporal expression patterns of other non-representative identified key genes are shown in ***Figure. S5.***

**References**

Ashburner, J., & Friston, K. J. (2009). Computing average shaped tissue probability templates. *Neuroimage, 45*(2), 333-341. doi:10.1016/j.neuroimage.2008.12.008

Brady, R. O., Jr., Gonsalvez, I., Lee, I., Ongur, D., Seidman, L. J., Schmahmann, J. D., . . . Halko, M. A. (2019). Cerebellar-Prefrontal Network Connectivity and Negative Symptoms in Schizophrenia. *Am J Psychiatry*, appiajp201818040429. doi:10.1176/appi.ajp.2018.18040429

Chen, J., Chen, R., Xue, C., Qi, W., Hu, G., Xu, W., . . . Zhang, X. (2022). Hippocampal-Subregion Mechanisms of Repetitive Transcranial Magnetic Stimulation Causally Associated with Amelioration of Episodic Memory in Amnestic Mild Cognitive Impairment. *J Alzheimers Dis, 85*(3), 1329-1342. doi:10.3233/JAD-210661

Chen, J., Ma, N., Hu, G., Nousayhah, A., Xue, C., Qi, W., . . . Zhang, X. (2020). rTMS modulates precuneus-hippocampal subregion circuit in patients with subjective cognitive decline. *Aging (Albany NY), 12*. doi:10.18632/aging.202313

First, M. B., Spitzer, R. L., Gibbon, M., & Williams, J. B. (2002). *Structured Clinical Interview for DSM-IV-TR Axis I Disorders, Research Version, Patient Edition. (SCID-I/P, Version 2.0)*: Biometrics Research. New York State Psychiatric Institute, New York, US.

Friston, K. J., Williams, S., Howard, R., Frackowiak, R. S., & Turner, R. (1996). Movement-related effects in fMRI time-series. *Magn Reson Med, 35*(3), 346-355. Retrieved from <http://www.ncbi.nlm.nih.gov/pubmed/8699946>

Power, J. D., Barnes, K. A., Snyder, A. Z., Schlaggar, B. L., & Petersen, S. E. (2012). Spurious but systematic correlations in functional connectivity MRI networks arise from subject motion. *Neuroimage, 59*(3), 2142-2154. doi:10.1016/j.neuroimage.2011.10.018

Van Dijk, K. R., Sabuncu, M. R., & Buckner, R. L. (2012). The influence of head motion on intrinsic functional connectivity MRI. *Neuroimage, 59*(1), 431-438. doi:10.1016/j.neuroimage.2011.07.044

Wang, X., Yao, S., Kirkpatrick, B., Shi, C., & Yi, J. (2008). Psychopathology and neuropsychological impairments in deficit and nondeficit schizophrenia of Chinese origin. *Psychiatry Res, 158*(2), 195-205. doi:10.1016/j.psychres.2006.09.007

Yan, C. G., Craddock, R. C., Zuo, X. N., Zang, Y. F., & Milham, M. P. (2013). Standardizing the intrinsic brain: towards robust measurement of inter-individual variation in 1000 functional connectomes. *Neuroimage, 80*, 246-262. doi:10.1016/j.neuroimage.2013.04.081

Yan, C. G., Wang, X. D., Zuo, X. N., & Zang, Y. F. (2016). DPABI: Data Processing & Analysis for (Resting-State) Brain Imaging. *Neuroinformatics, 14*(3), 339-351. doi:10.1007/s12021-016-9299-4

Zhou, C., Yu, M., Tang, X., Wang, X., Zhang, X., Zhang, X., & Chen, J. (2019). Convergent and divergent altered patterns of default mode network in deficit and non-deficit schizophrenia. *Prog Neuropsychopharmacol Biol Psychiatry, 89*, 427-434. doi:10.1016/j.pnpbp.2018.10.012

***Table S1 The names and abbreviations of the regions of interest (ROIs) in AAL3v1 atlas***

| **NO.** | **Regions** | **Abbr.** | **NO.** | **Regions** | **Abbr.** |
| --- | --- | --- | --- | --- | --- |
| 1, 2 | Precentral gyrus | PreCG | 91,92 | Temporal pole: middle | TPOmid |
| 3, 4 | Superior frontal gyrus, dorsolateral | SFGdor | 93,94 | Inferior temporal gyrus | ITG |
| 5, 6 | Middle frontal gyrus | MFG | 95,96 | Crus I of cerebellar hemisphere | CERCRU1 |
| 7, 8 | Inferior frontal gyrus, opercular part | IFGoperc | 97,98 | Crus II of cerebellar hemisphere | CERCRU2 |
| 9, 10 | Inferior frontal gyrus, triangular part | IFGtriang | 99,100 | Lobule III of cerebellar hemisphere | CER3 |
| 11, 12 | IFG pars orbitalis | IFGorb | 101,102 | Lobule IV, V of cerebellar hemisphere | CER4_5 |
| 13, 14 | Rolandic operculum | ROL | 103,104 | Lobule VI of cerebellar hemisphere | CER6 |
| 15, 16 | Supplementary motor area | SMA | 105,106 | Lobule VIIB of cerebellar hemisphere | CER7b |
| 17, 18 | Olfactory cortex | OLF | 107,108 | Lobule VIII of cerebellar hemisphere | CER8 |
| 19, 20 | Superior frontal gyrus, medial | SFGmed | 109,110 | Lobule IX of cerebellar hemisphere | CER9 |
| 21, 22 | Superior frontal gyrus, medial orbital | PFCventmed | 111,112 | Lobule X of cerebellar hemisphere | CER10 |
| 23, 24 | Gyrus rectus | REC | 113 | Lobule I, II of vermis | VER1_2 |
| 25, 26 | Medial orbital gyrus | OFCmed | 114 | Lobule III of vermis | VER3 |
| 27, 28 | Anterior orbital gyrus | OFCant | 115 | Lobule IV, V of vermis | VER4_5 |
| 29, 30 | Posterior orbital gyrus | OFCpost | 116 | Lobule VI of vermis | VER6 |
| 31, 32 | Lateral orbital gyrus | OFClat | 117 | Lobule VII of vermis | VER7 |
| 33, 34 | Insula | INS | 118 | Lobule VIII of vermis | VER8 |
| 35, 36 | Anterior cingulate & paracingulate gyri | ACG | 119 | Lobule IX of vermis | VER9 |
| 37, 38 | Middle Cingulate & paracingulate gyri | MCC | 120 | Lobule X of vermis | VER10 |
| 39, 40 | Posterior cingulate gyrus | PCC | 121,122 | Thalamus, Anteroventral Nucleus | tAV |
| 41, 42 | Hippocampus | HIP | 123,124 | Lateral posterior | tLP |
| 43, 44 | Parahippocampal gyrus | PHG | 125,126 | Ventral anterior | tVA |
| 45, 46 | Amygdala | AMYG | 127,128 | Ventral lateral | tVL |
| 47, 48 | Calcarine fissure & surrounding cortex | CAL | 129,130 | Ventral posterolateral | tVPL |
| 49, 50 | Cuneus | CUN | 131,132 | Intralaminar | tIL |
| 51, 52 | Lingual gyrus | LING | 133,134 | Reuniens | tRe |
| 53, 54 | Superior occipital gyrus | SOG | 135,136 | Mediodorsal medial magnocellular | tMDm |
| 55, 56 | Middle occipital gyrus | MOG | 137,138 | Mediodorsal lateral parvocellular | tMDl |
| 57, 58 | Inferior occipital gyrus | IOG | 139,140 | Lateral geniculate | tLGN |
| 59, 60 | Fusiform gyrus | FFG | 141,142 | Medial Geniculate | tMGN |
| 61, 62 | Postcentral gyrus | PoCG | 143,144 | Pulvinar anterior | tPuA |
| 63, 64 | Superior parietal gyrus | SPG | 145,146 | Pulvinar medial | tPuM |
| 65, 66 | Inferior parietal gyrus | IPG | 147,148 | Pulvinar lateral | tPuL |
| 67, 68 | Supramarginal gyrus | SMG | 149,150 | Pulvinar inferior | tPuI |
| 69, 70 | Angular gyrus | ANG | 151,152 | Anterior cingulate cortex, subgenual | ACCsub |
| 71, 72 | Precuneus | PCUN | 153,154 | Anterior cingulate cortex, pregenual | ACCpre |
| 73, 74 | Paracentral lobule | PCL | 155,156 | Anterior cingulate cortex, supracallosal | ACCsup |
| 75, 76 | Caudate nucleus | CAU | 157,158 | Nucleus accumbens | Nacc |
| 77, 78 | Lenticular nucleus, putamen | PUT | 159,160 | Ventral tegmental area | VTA |
| 79, 80 | Lenticular nucleus, pallidum | PAL | 161,162 | Substantia nigra, pars compacta | SNpc |
| 81, 82 | Thalamus | THA | 163,164 | Substantia nigra, pars reticulata | SNpr |
| 83, 84 | Heschl gyrus | HES | 165,166 | Red nucleus | RedN |
| 85, 86 | Superior temporal gyrus | STG | 167,168 | Locus coeruleus | LC |
| 87, 88 | Temporal pole: superior | TPOsup | 169 | Raphe nucleus, dorsal | RapheD |
| 89, 90 | Middle temporal gyrus | MTG | 170 | Raphe nucleus, median | RapheM |

***Table S2* *The detailed options in the processing gene expression data code for the four parcellations***

| **Parameters and options Annotation** | **Parameters and options Annotation** |
| --- | --- |
| options.ExcludeCBandBS = true | Samples in brainstem and cerebellum are excluded based on their labels provided by the AHBA |
| options.useCUSTprobes = true | Custom and Agilent probes are used |
| options.updateProbes =’ reannotator’ | Reannotator software is used to update annotations from probes to genes |
| options.probeSelections = {‘RNAseq’} | RNA-seq data are used for probe selection |
| options.parcellations = {‘HCP’}  /options.parcellations = {‘Schaefer300’}  /options.parcellations = {‘Schaefer500’}  /options.parcellations = {‘Schaefer1000’} | HCP template (180 nodes per hemisphere) is used for brain parcellation; Schaefer300, Schaefer500, and Schaefer1000 parcellations (150, 250, 500 nodes per hemisphere, respectively) are used for brain parcellation, respectively. |
| options.distanceThreshold = 2 | The distance threshold is defined as 2 mm when assigning samples to brain regions |
| options.signalThreshold = 0.5 | Probes that do not exceed background signals in at least 50% of samples are excluded |
| options.divideSamples = ‘listCortex’ | Samples are divided into cortex and subcortex based on re-defined list of brain regions |
| options.excludeHippocampus = false | Hippocampal samples are not excluded during sample assignment |
| options.VARfilter = false | Variance based filtering is not performed |
| options.VARscale = ‘normal’ | Variance based filtering is performed on non-log2 transformed data |
| options.VARperc = 50 | We exclude 50% probes with the lower variance |
| options.RNAseqThreshold = 0.2 | Probes with low correlations (r<0.2) between expression values measured by microarray and RNAseq are excluded |
| options.RNAsignThreshold = false | No significance threshold is used for expression correlation between RNAseq and microarray |
| options.correctDistance = false | Distance correction is not used for accounting for spatial effects |
| options.calculateDS = true | Differential stability is calculated for each gene |
| options.distanceCorrection = ‘Euclidean’ | Distances between samples are evaluated as Euclidean distances between sample coordinates |
| options.Fit = {‘exp’} | Exponential fit is used to estimate the relationship between correlated gene expression and distance |
| options.normaliseWhat = ‘Lcortex’ | Only expression data from the left cortex are used (6 subjects) |
| options.normMethod = ‘scaledRobustSigmoid’ | Scaled outlier-robust sigmoid normalization method is used |
| options.percentDS = 100 | The differential stability values of all genes (100%) are calculated |
| options.saveOutput = true | The output of the analysis is saved |
| options.normaliseWithinSample = true | The expression values are normalized before within gene normalization |
| options.normaliseWithinSample = ‘meanSamples’ | When multiple samples are available for a given brain region, the mean expression value of all samples is calculated to summarize the expression vector. The “weight” of each sample in that region is equal |

***Table S3 Periods of human development and adulthood as defined in*** ***spatio-temporal dynamics of the human brain transcriptome***

| **Period** | **Description** | **Age** |
| --- | --- | --- |
| **1** | Embryonic | 4PCW≤Age<8PCW |
| **2** | Early fetal | 8PCW≤Age<10PCW |
| **3** | Early fetal | 10PCW≤Age<13PCW |
| **4** | Early mid-fetal | 13PCW≤Age<16PCW |
| **5** | Early mid-fetal | 16PCW≤Age<19PCW |
| **6** | Late mid-fetal | 19PCW≤Age<24PCW |
| **7** | Late fetal | 24PCW≤Age<38PCW |
| **8** | Neonatal and early infancy | 0M (birth)≤Age<6M |
| **9** | Late infancy | 6M≤Age<12M |
| **10** | Early childhood | 1 Y≤Age<6Y |
| **11** | Middle and late childhood | 6 Y≤Age<12 Y |
| **12** | Adolescence | 12 Y≤Age<20 Y |
| **13** | Young adulthood | 20 Y≤Age<40 Y |
| **14** | Middle adulthood | 40 Y≤Age<60 Y |
| **15** | Late adulthood | 60 Y≤Age |

**M, postnatal months; PCW, post-conceptional weeks; Y, postnatal years**

***Table S4 The full name of the identified genes in DS and NDS***

| **Abbreviated gene name** | **Full name** |
| --- | --- |
| **Positive overlap genes between DS and NDS** | |
| **SCN1B** | sodium channel, voltage-gated, type I, beta subunit |
| **ASB13** | ankyrin repeat and SOCS box containing 13 |
| **CADPS2** | Ca++-dependent secretion activator 2 |
| **IFFO1** | intermediate filament family orphan 1 |
| **SHD** | Src homology 2 domain containing transforming protein D |
| **CCNI** | cyclin I |
| **GLCCI1** | glucocorticoid induced transcript 1 |
| **GPR158** | G protein-coupled receptor 158 |
| **IGFBP2** | insulin-like growth factor binding protein 2, 36kDa |
| **KLF9** | Kruppel-like factor 9 |
| **LAPTM4B** | lysosomal protein transmembrane 4 beta |
| **ZMAT4** | zinc finger, matrin-type 4 |
| **Negative overlap genes between DS and NDS** | |
| **DOK6** | docking protein 6 |
| **DPYSL3** | dihydropyrimidinase-like 3 |
| **EFNB3** | ephrin-B3 |
| **KCNN3** | potassium intermediate/small conductance calcium-activated channel, subfamily N, member 3 |
| **RAB27B** | RAB27B, member RAS oncogene family |
| **LRRC3B** | leucine rich repeat containing 3B |
| **NKAIN4** | Na+/K+ transporting ATPase interacting 4 |
| **PPM1M** | protein phosphatase, Mg2+/Mn2+ dependent, 1M |
| **SLC16A2** | solute carrier family 16, member 2 (thyroid hormone transporter) |
| **EDNRB** | endothelin receptor type B |

***Figure S1 The results of significant different FC between DS and NDS***

***
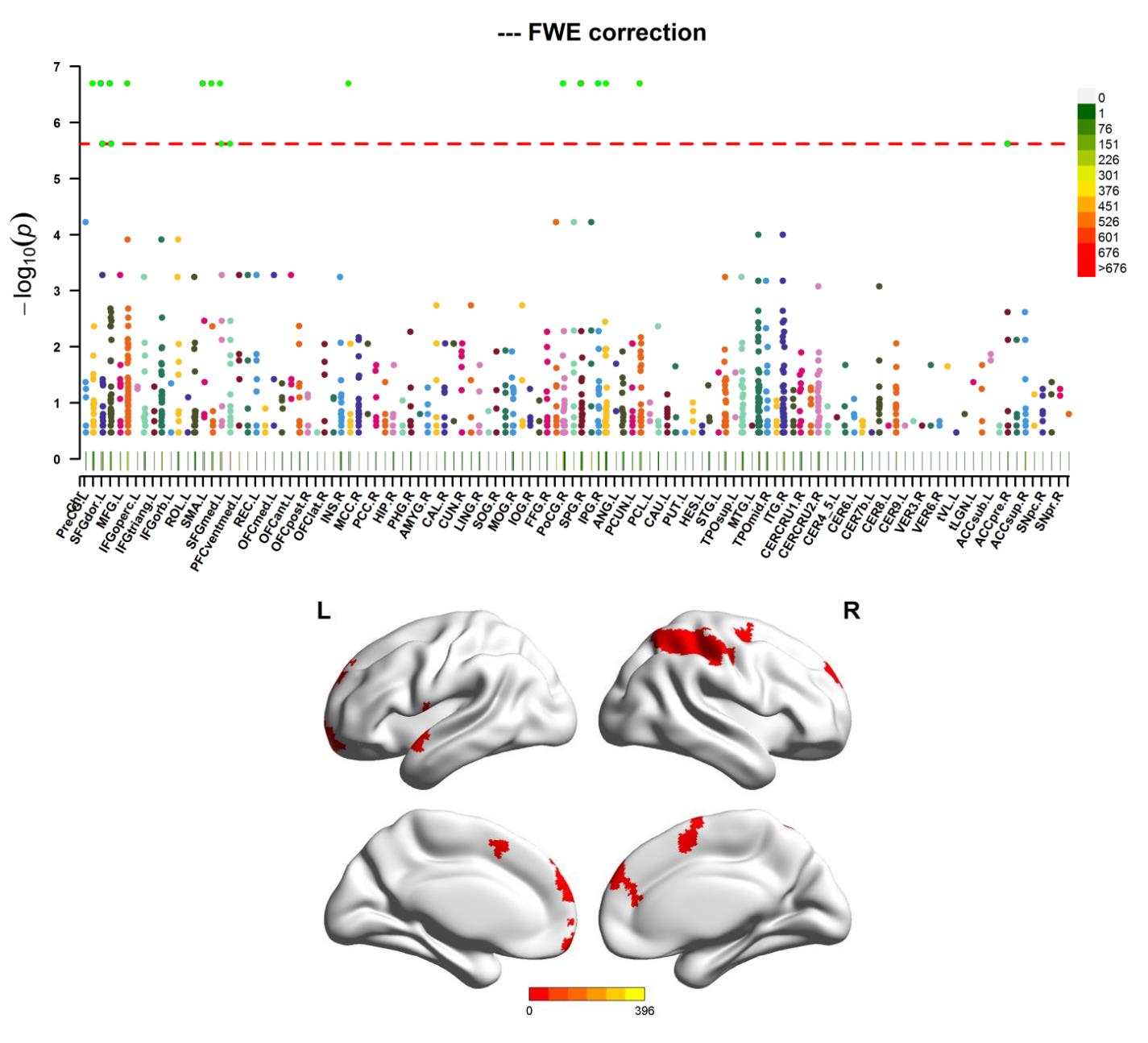
***

***Figure S2 The results of LDA for classification of DS and NDS***

***
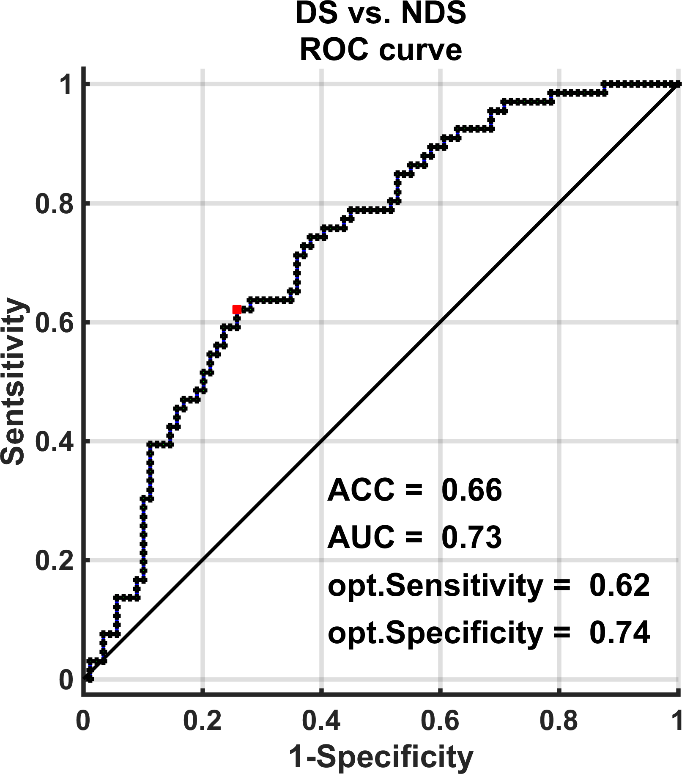
***

***Figure S3 The results of SVM for classification of DS and NDS***

***
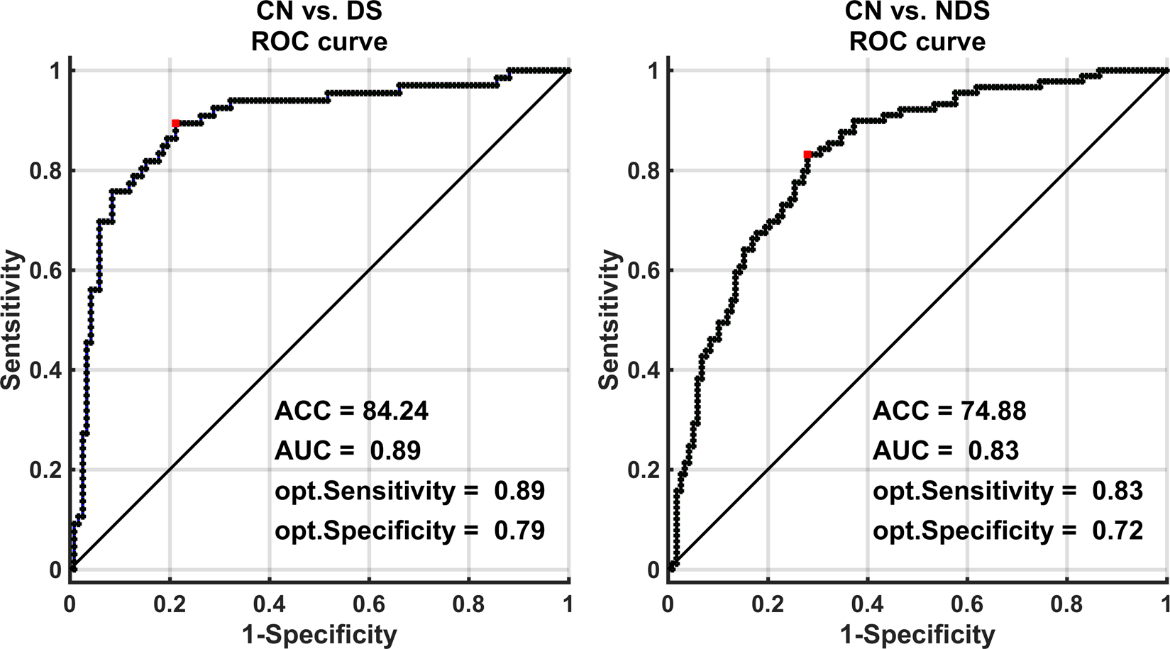
***

***Figure S4 The results of WGCNA with different parameter sets***

***
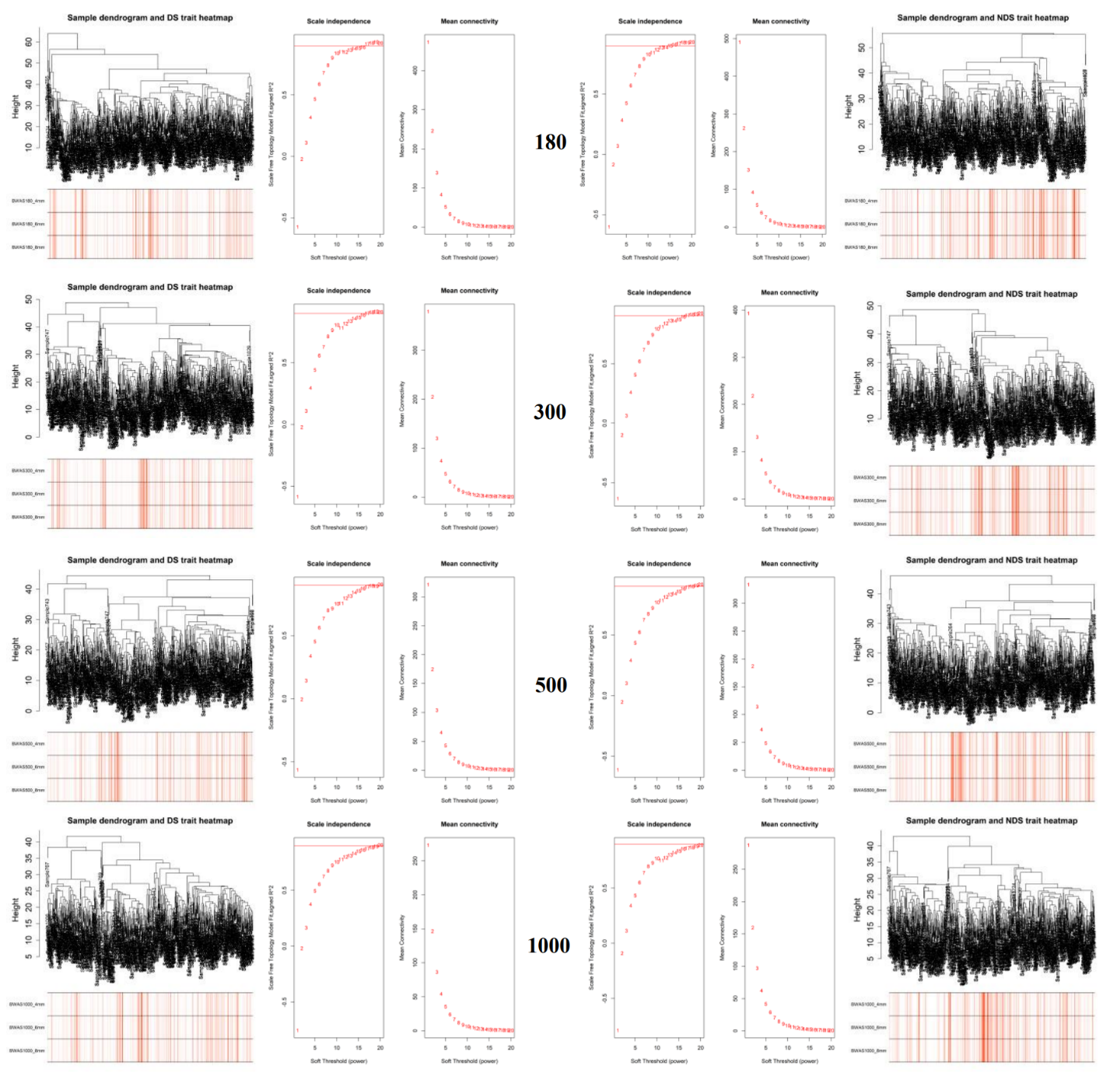
***

***Figure S5.1* *The temporal expression patterns of negative overlap genes***

**
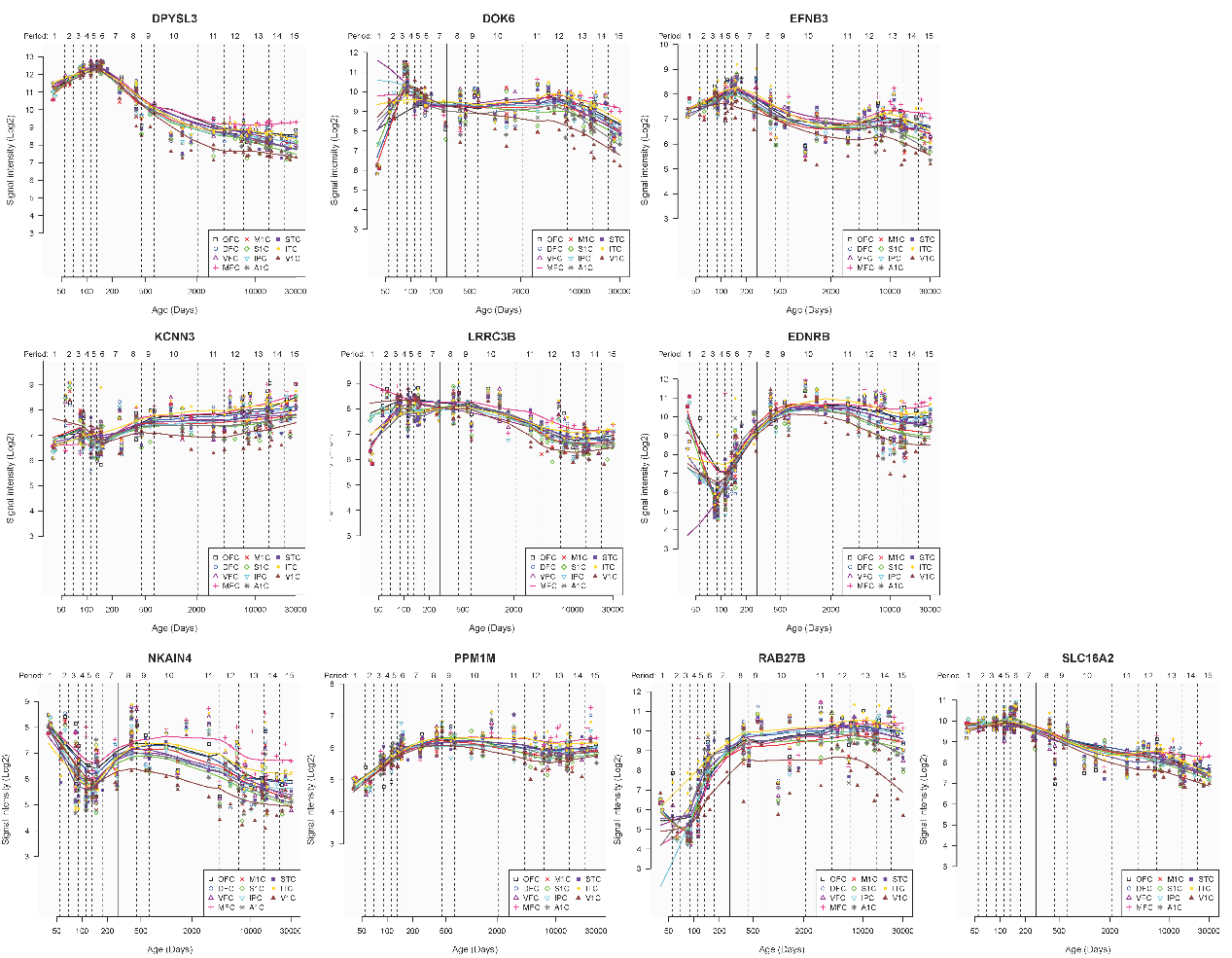
**

***Figure S5.2* *The temporal expression patterns of positive overlap genes***

**
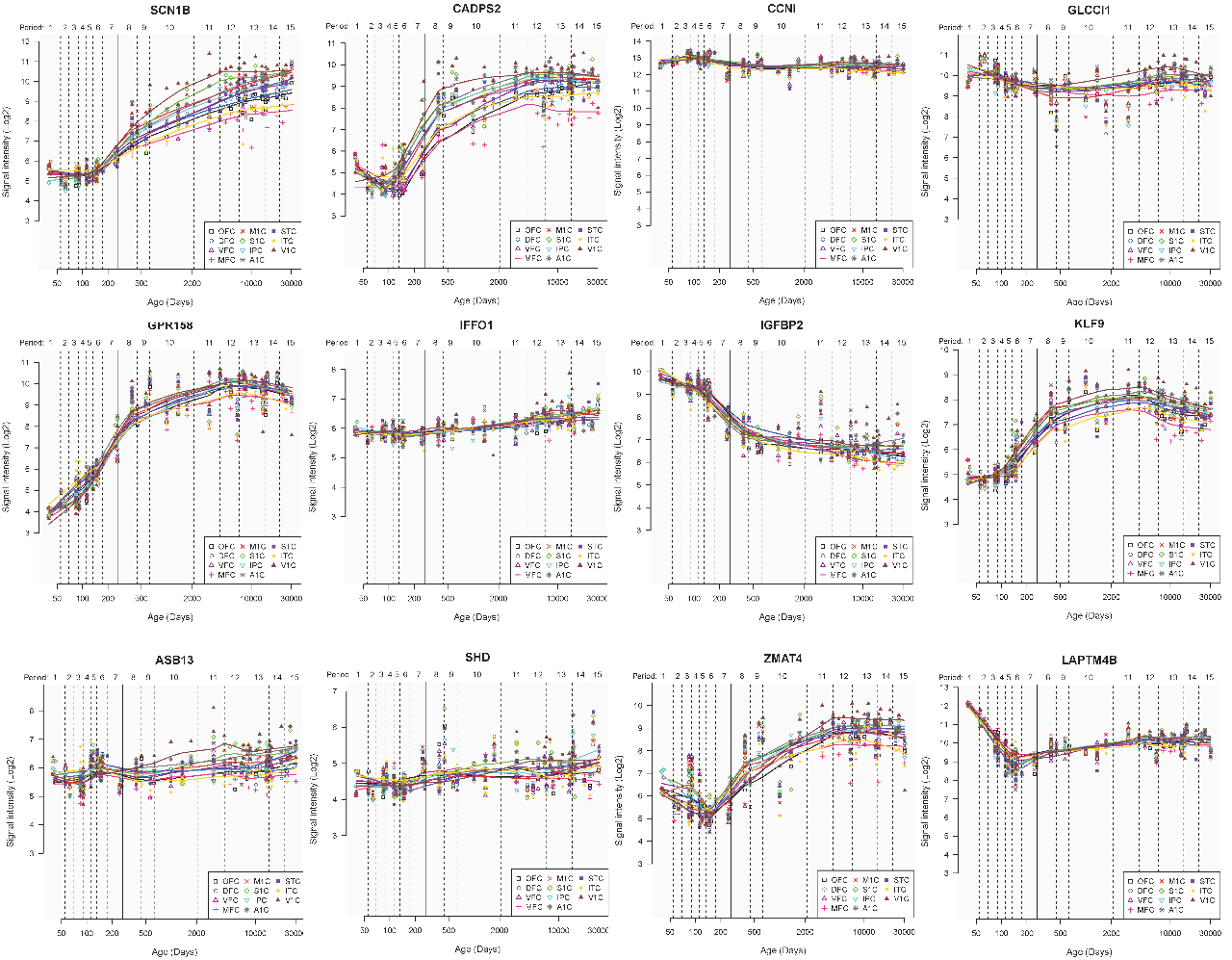
**
